# Fc-enhanced anti-CCR6 antibody elicits robust therapeutic effects across multiple autoimmune diseases

**DOI:** 10.3389/fimmu.2025.1728419

**Published:** 2026-01-09

**Authors:** Md Jahangir Alam, Yu-Anne Yap, Caroline Ang, Liang Xie, Hillary L. Shane, Charles R. Mackay, Remy Robert

**Affiliations:** 1 Department of Physiology, Biomedicine Discovery Institute, Monash University, Clayton, VIC, Australia; 2 Department of Microbiology, Biomedicine Discovery Institute, Monash University, Clayton, VIC, Australia; 3 Dragonfly Therapeutics, Waltham, MA, United States

**Keywords:** arthritis, CCR6, IL-17A, inflammation, monoclonal antibody (mAb), psoriasis, scleroderma, Th17 cells

## Abstract

Activation of the chemokine receptor CCR6 orchestrates the trafficking of IL-17-producing pathogenic immune cells to the sites of inflammation, thus contributing to the development of numerous inflammatory and autoimmune diseases. As such, CCR6 has emerged as a promising therapeutic target for treating Th17-mediated inflammatory disorders. In this study, we employed a targeted strategy, which we termed ‘immunological surgery’, using an Fc-engineered anti-human CCR6 monoclonal antibody (αhCCR6 DLE-mut mAb) designed to engage effector mechanisms against CCR6^+^ immune cells and deplete them. We evaluated the therapeutic efficacy of this approach in preclinical mouse models of representative autoimmune conditions, including scleroderma, psoriasis, and rheumatoid arthritis. Selective targeting of CCR6^+^ cells with αhCCR6 DLE-mut mAb exhibited remarkable efficacy in reducing established inflammation across all disease models. In a bleomycin-induced scleroderma model, αhCCR6 mAb treatment markedly reduced dermal thickening and attenuated scleroderma-associated lung inflammation and fibrosis. In the imiquimod-induced psoriasis model, administration of αhCCR6 mAb led to significant reductions in skin thickening, epidermal hyperplasia, and dermal immune cell infiltration. Similarly, in the collagen-induced arthritis (CIA) model, αhCCR6 mAb treatment significantly alleviated all signs of joint inflammation. Thus, our findings demonstrated that CCR6-targeted therapy could be a promising and effective approach for the treatment of Th17-mediated inflammatory disorders. Moreover, we believe this approach may overcome the challenge of chemokine receptor redundancy by leveraging receptor-specific signatures to eliminate pathogenic leukocyte subsets with high precision.

## 1 Introduction

The migration of leukocytes to inflammation sites in response to various chemotactic stimuli allows the immune system to fight an aggression, defend against invading pathogens, and maintain normal homeostasis. While this process is essential for host defense and immune homeostasis, an excessive or chronic influx of immune cells into specific tissues can lead to various immune-mediated inflammatory pathologies, such as autoimmune diseases and chronic inflammatory disorders. Consequently, the selective targeting of chemokine receptors to inhibit the migration of rogue immune cells has been considered an attractive therapeutic strategy to treat inflammation associated with inflammatory and autoimmune diseases.

For instance, the chemokine receptor CCR6, which is predominantly expressed on IL-17 and IL-22-producing T-cells, including Th17 cells (1, 2) and subsets of B-cells (3, 4), has been implicated in numerous inflammatory and autoimmune conditions and facilitates the migration of pathogenic immune cells to the sites of inflammation (5–7). In murine models of psoriasis, a distinct immune cell population expressing low levels of γδ T-cell receptor (TCRγδ) and high levels of CCR6 serves as a significant source of IL-17 (8–11), contributing to the initiation and maintenance of skin inflammation (10, 12, 13). Similarly, in humans, dermal γδ T cells secrete IL-17, which plays a critically pathogenic role in skin inflammatory reactions (8).

In systemic sclerosis, both Th17 cells and IL-17 have been implicated in disease progression and fibrosis in mouse models (14, 15), and IL-17^-/-^ mice exhibit resistance to the disease (15). Elevated levels of Th17 cells and IL-17 have been observed in patients with scleroderma (16–20). Moreover, IL-17 has been shown to promote fibroblast activation and collagen production in scleroderma patients (21), further supporting its critical involvement with the development of fibrosis.

In rheumatoid arthritis (RA) patients, CCR6^+^ cells are elevated in the inflamed synovium and peripheral blood (22–24). In RA joints, myeloid cells exhibit increased secretion of CCL20 and facilitate the recruitment of CCR6^+^ Th17 cells, thereby sustaining joint inflammation (1). Fibroblast-like synoviocytes also produce CCL20, further contributing to local inflammation by attracting Th17 cells into the synovium (25).

The pathogenic role of CCR6 has been validated in multiple preclinical models of autoimmune and inflammatory disorders, through genetic deletion or pharmacological blockade (10, 26–28). Previously, we and others have shown that CCR6 blockade using mAbs and/or small molecules was effective in treating psoriasis and experimental autoimmune encephalomyelitis (EAE) (1, 13). However, despite the well-documented involvement of chemokine receptors in many inflammatory diseases, the redundancy of the chemokine system has been thought to be a major reason for the failure of drug development in this area. To overcome this challenge, we developed a novel therapeutic strategy based on the targeting of CCR6^+^ cells using a Fc-engineered anti-human CCR6 mAb (αhCCR6 DLE-mut mAb). This approach, which we term ‘immunological surgery’, is designed to engage Fc-dependent effector functions against pathogenic immune subsets defined by their chemokine receptor expression, rather than merely blocking receptor signaling. We evaluated the efficacy of αhCCR6 DLE-mut mAb in human CCR6 knock-in (hCCR6 KI) mice across three representative models of IL-17-driven autoimmunity: bleomycin-induced scleroderma, imiquimod-induced psoriasis, and collagen-induced arthritis (CIA). Given the central role of CCR6 in mediating the migration and effector functions of IL-17-producing Th17 cells, we hypothesized that targeting CCR6^+^ cells using Fc-enhanced mAb would yield broad therapeutic benefit across multiple autoimmune contexts.

Our results demonstrate that treatment with αhCCR6 mAb significantly attenuates disease severity and immune cell infiltration in all three models, highlighting the potential of this CCR6-targeting Fc-enhanced antibody strategy as a promising and viable immunotherapy with translational relevance across multiple autoimmune disease contexts. Moreover, this approach could be potentially extended to other chemokine receptor signatures for the targeted depletion of specific pathogenic cell subsets through antibody-mediated interventions and may overcome the limitations posed by chemokine system redundancy in drug development.

## 2 Materials and methods

### 2.1 Mice

Human CCR6 knock-in (hCCR6-Tg/mCCR6^-/-^) mice on a C57BL/6 background were generated using CRISPR/Cas9-based protocol as previously reported (29). hCCR6-Tg/mCCR6^-/-^ female mice between 8 and 10 weeks of age were obtained from the Monash Animal Research Platform, Monash University, Victoria, Australia. Mice were maintained under specific pathogen-free conditions on a 12/12-hour light/dark cycle and had free access to food and water. All animal care and experimental procedures used in this study were approved by the Animal Ethics Committee of Monash University.

### 2.2 Generation and engineering of αhCCR6 mAb

The αhCCR6 mAb (clone# 6H12) used throughout this study was isolated and humanized as previously described (13). The αhCXCR2 antibody (clone# TAHX2) was used as an isotype control (30).

To produce the DLE depleting variants (DLE Fc- S239D/A330L/I332E) human IgG1, standard mutagenesis techniques were used. The DLE mutations increase the affinity of the Fc for both human and mouse FcγR and thus increase ADCC and ADCP activities. The sequences were confirmed on both strands by DNA sequencing.

The variable genes were amplified by PCR and cloned into a mammalian expression vector containing the human kappa and human heavy chain IgG1 Fc DLE constant regions. Recombinant DNA was transfected into CHO-DG44 cells using the AMAXA nucleofactor device (Lonza) according to the manufacturer’s protocol. The transfected CHO cells were grown and maintained in a serum-free medium (Invitrogen) for antibody production. The antibodies were purified from the culture supernatant by affinity chromatography on a ProsepvA protein A column (Millipore). The bound mAbs were eluted with 0.2 M glycine/1 M NaCl, pH 3.0 into a neutralizing solution of 1 M Tris, pH 8.0, and then the buffer was exchanged against phosphate-buffered saline (PBS). Purified antibodies were endotoxin-free as determined by a chromogenic LAL assay (GenScript).

### 2.3 Antibody-dependent cell-mediated cytotoxicity assay

The ADCC activity of αhCCR6 mAbs was assessed by flow cytometry, following established protocols (13, 31). Briefly, hCCR6-expressing L1.2 target cells were labeled with the membrane dye PKH26 to allow discrimination from effector cells during co-culture. Labeled target cells were washed 3 times and resuspended at 2 × 10^6^ cells/mL in culture medium and seeded into round-bottom 96-well plates (5 × 10^4^ cells/well in 25 μL). Cells were preincubated for 30 minutes at 37°C with serial dilutions 1:4, starting from 2 μg/mL of αhCCR6 DLE-mut, αhCCR6 Fc-Null, or human IgG1 isotype control mAbs.

Human PBMCs were isolated from heparinized whole blood of healthy donors via Ficoll-Paque density gradient centrifugation. NK cells were subsequently isolated using MACS CD56 microbeads and positive selection columns (Miltenyi Biotec), according to the manufacturer’s instructions. Purified NK cells (2 × 10^5^/well) were added to the antibody-treated target cells at an effector-to-target (E:T) ratio of 4:1 and incubated for 3 hours at 37°C in the presence of 10% heat-inactivated human serum. Following incubation, cells were transferred to microtiter tubes containing 10 μM of TO-PRO-3 iodide (Thermo Fisher Scientific) to identify dead cells. Samples were acquired using a BD FACSymphony™ flow cytometer and analyzed using FlowJo software v10 (FlowJo, LLC).

### 2.4 Bleomycin-induced scleroderma

Mice were anaesthetized using 4% isoflurane in 2L/minute of oxygen. The backs of anaesthetized mice were shaved with an electric clipper and then treated with depilatory cream to remove hair. The next day, mice were subcutaneously (s.c.) injected with 100 μL of Bleomycin (BLM) (Millipore Sigma) solution (1 mg/mL in PBS) at a single location on the shaved backs of mice for 4 weeks using a 27-gauge needle. The exact amount of PBS was injected as a control. Dorsal skin thickness was measured every other day just before s.c. injection of BLM with a digital thickness gauge, and the change in percentage was calculated compared to the thickness on day 0. For the therapeutic regimen, mice were treated with BLM until their dorsal skin thickening (~15%) exhibited symptoms of the disease and the onset of fibrotic signs (32, 33) (on day 8). A loading dose (25 mg/kg of body weight) of either an αhCCR6 or an isotype control mAbs was administered intraperitoneally (i.p.), followed by maintenance doses (5 mg/kg, i.p.) twice a week. This antibody regimen was chosen based on our published study using an anti-CXCR2 mAb (30). Mice were evaluated daily by an observer blind to the treatment groups. Back skin redness (erythema) and the presence of scales (scaling) of the skin were scored using a semiquantitative scoring system from 0 to 4 based on their external physical appearance: 0 = no skin abnormalities, 1 = slight, 2 = moderate, 3 = marked, and 4 =severe. On day 29, mice were humanely killed by CO_2_ asphyxiation at a controlled flow rate of 50% of the chamber volume per minute. Dorsal skin, skin-draining lymph nodes (LNs), spleen, lung, and lung-draining LNs were subsequently harvested for assessment of cellular infiltration and histological analysis.

### 2.5 Imiquimod-induced psoriasis

Mice were anaesthetized using 4% isoflurane in 2L/minute of oxygen. Their backs were shaved with an electric clipper and then treated with depilatory cream to remove hair. On the next day, 20 mg IMQ cream 5% (Aldara; 3M Pharmaceuticals) or vaseline (as a control cream) was applied daily to the back (dorsal) skin for 7 consecutive days. Dorsal skin thickness was measured every day just before the application of IMQ cream using a digital thickness gauge, and the change in percentage was calculated compared to the thickness on day 0. For the therapeutic regimen, mice were treated with IMQ cream until dorsal skin thickening (~30%) indicated disease progression (on day 3). Mice were then administered a loading dose (25 mg/kg of body weight, i.p.) of either an αhCCR6 or an isotype control (which one), followed by maintenance doses (5 mg/kg, i.p.) every other day for a week. Mice were evaluated daily by an observer blind to the treatment groups. Dorsal skin erythema and scaling were scored using a semiquantitative scoring system from 0 to 4 based on their external physical appearance: 0 = no skin abnormalities, 1 = slight, 2 = moderate, 3 = marked, and 4 = severe. On day 9, mice were humanely killed by CO_2_ asphyxiation at a controlled flow rate of 50% of the chamber volume per minute. Following euthanasia, dorsal skin, spleen, and skin-draining LNs were collected for evaluation of cellular infiltration and histological changes.

### 2.6 Collagen-induced arthritis

Mice were anaesthetized using 4% isoflurane in 2L/minute of oxygen, and immunized s.c. at two sites at the base of the tail with a 100 µL emulsion of 100 µg Chicken type II collagen (CII, Sigma) and 200 µg *Mycobacterium tuberculosis* in Complete Freund’s Adjuvant (Chondrex) in equal volumes, as previously described (34). On Day 21 post-immunization, mice were boosted with 100 µg of CII emulsified with Incomplete Freund’s adjuvant (Sigma). Mice were evaluated and scored for arthritis three times a week from Day 18 post-immunization. Arthritis severity was scored on a scale of 0 to 4, as follows: 0 = no evidence of erythema and swelling; 1 = one or two toes inflamed and swollen with no apparent swelling of the paw or ankle; 2 = Three or more toes inflamed and swollen but no paw swelling or mild swelling of entire paw; 3 = swelling of entire paw; and 4 = severe swelling of entire paw or ankylosis of the paw. Arthritis severity was determined as the sum of scores for all four legs. When mice exhibited swelling in ankle joints and front paws with an average cumulative clinical score of 4 (day 41), mice were treated with eight i.p injections of 5 mg/kg of body weight with the αhCCR6 or isotype control mAbs, every other day for 3 weeks. On day 60, mice were humanely killed by CO_2_ asphyxiation at a controlled flow rate of 50% of the chamber volume per minute. Thereafter, blood, ankle joints, and popliteal draining LNs were harvested for analysis of CII-specific IgG antibodies, cellular infiltration, and histology.

### 2.7 Histological evaluation and quantification

Following euthanasia through CO_2_ asphyxiation, lung, dorsal skin, and ankle samples were excised, collected and fixed in 10% neutral buffered formalin (NBF) for 24 hours. Tissues were processed, followed by paraffin embedding and sectioning at 4 µm. Lung and skin sections were stained with hematoxylin and eosin (H&E) and evaluated at 20× magnification to quantify the dermal thickness using ImageJ software (National Institutes of Health). The percentage of fibrosis in lung tissue sections stained with Masson’s trichrome (MT) was quantified using ImageJ software. Lung and skin tissue sections were stained with Picrosirius red (PSR) to determine collagen deposition. MT staining was performed on the skin and lung sections to analyze fibrosis using a numerical fibrosis scoring scale of the modified Ashcroft score (35, 36), a score of 0 was considered as no fibrosis, 1 as minimal, 2 as mild, 3 as moderate, and 4 as severe fibrosis. All sections were scored independently by an investigator in a blinded manner.

For both scleroderma and psoriasis skin tissue sections, the dermal thickness was measured at five different locations on the same slide, and the average value was calculated to represent the findings for each tissue sample. Ankle sections were stained with H&E and Safranin O to examine cell infiltration and cartilage destruction.

### 2.8 Mouse tissue processing and single-cell preparation

For skin single-cell suspension, dorsal skin pieces were taken from the same anatomical region across animals. We excised a 1.0 × 1.0 cm full-thickness dorsal skin piece and trimmed subcutaneous fat. Thus, tissue size and weight were similar across animals through uniform anatomical dissection, ensuring comparability of absolute cell counts. Skin samples were minced with scissors and digested with 0.2 mg/mL of Liberase TL (Roche Diagnostics) and 0.5 mg/mL of DNAse (Roche Diagnostics) in RPMI for 1 hour at 37 °C with agitation. After the digestion, the cell suspension and the remaining tissue fragments were passed through a 70 μm cell strainer and washed with cold RPMI-1640 containing 5 mM EDTA. The cell suspension was filtered through 100 μm and then 40 μm nylon mesh filters, and washed with cold FACS buffer (PBS containing 2% FCS and 4 mM EDTA).

For mouse lung single-cell suspensions, lungs were perfused with PBS, minced with scissors in RPMI-1640 medium (Gibco by Life Technologies) with 10% of FCS, and digested with 0.5 mg/mL of Collagenase Type IV (Gibco by Life Technologies) and 1 µg/mL of DNase I (Roche Diagnostics) for 45 minutes at 37 °C under agitation. After the digestion, the cell suspension and the remaining tissue fragments were passed through a 70 μm cell strainer and washed with cold RPMI-1640 containing 5 mM of EDTA. Red blood cells were depleted with a lysis buffer and washed with cold PBS. For further purification, cell pellets were resuspended in 4 mL of 40% isotonic Percoll solution and centrifuged at 1000g for 20 mins at room temperature (RT) without using the brake. The cell pellets were then washed with cold PBS.

For single-cell suspensions harvested from the ankle joints, bone marrow cells were initially flushed out using a 27-gauge needle filled with 1 mL RPMI 1640. Ankles were then chopped into 3 to 4 mm chunks and digested with 2.4 mg/mL Hyaluronidase (Sigma) and 1 mg/mL Collagenase Type VIII (Sigma) in RPMI-1640 containing 10% BCS for 1 h at 37°C under agitation. Following digestion, the cell suspension was filtered through a 70 µm cell strainer and washed with cold FACS buffer to yield a single-cell suspension.

For mouse splenic cell suspensions, spleens were mechanically disrupted in cold PBS and passed through a 70 μm strainer. Cells were then subjected to red blood cell lysis and washed with cold PBS. For draining LNs, the LNs were mechanically disrupted, passed through a 70 μm cell strainer, and washed with cold FACS buffer to obtain a single-cell suspension.

### 2.9 Measurement of anti-CII antibodies

Plasma concentrations of anti-CII autoantibodies (Abs) were measured by ELISA. Briefly, a 96-well microtiter plate (NUNC MaxiSorp) was coated overnight at 4°C with 2 µg/mL chicken collagen (Sigma) in PBS. The plate was then washed with PBS Tween 0.05% (Amresco) and blocked in 2% BSA in PBS for 1 hour at RT. After incubation, the plates were washed with PBS Tween 0.05%, and diluted standards or plasma samples were added to the wells and incubated at RT for 2 hours. The plates were washed with PBS Tween 0.05% and HRP-conjugated anti-mouse IgG, IgG1, IgG2b or IgG2c (1:1000 dilution in PBS) was added and incubated for 1 hour at RT. TMB substrate (Thermo Scientific) was then added to each well and incubated for 15 mins. After incubation, the reaction was stopped with 0.5M H_2_SO_4_, and absorbance was measured at 450 nm and 540 nm. The final values were obtained by subtracting the 540 nm readings from the 450 nm readings. Diluted isotype control pooled plasma samples were used to generate the standard calibration curve, with the highest optical density (OD) value set to 1 arbitrary unit (AU). Data were analyzed using a four-parameter logistic (4PL) function for curve fitting and quantification.

### 2.10 Flow cytometry

Cells were suspended in FACS buffer and treated with mouse FcR blocking reagent (Miltenyi Biotech) for 10 mins at RT to prevent non-specific antibody binding during cell staining procedures. For myeloid cells analysis in the scleroderma model, cells were stained with surface marker-specific following antibodies in the dark at 4°C for 30 mins: anti-mouse CD45 (clone 30-F11, BD Pharmingen); anti-mouse CD11b (clone M1/70, BD Pharmingen); anti-mouse Ly6G (clone 1A8, BioLegend); anti-mouse Siglec-F (clone E50-2440, BD Pharmingen); anti-mouse CD11c (clone HL3, BD Pharmingen); anti-mouse CD19 (clone 1D3, BD Pharmingen), anti-mouse CD45R (clone RA3-6B2, BD Horizon); anti-mouse PDCA-1 (clone eBio927, eBioscience). Dead cells were excluded using 7-AAD (BD Pharmingen).

For T cell analysis in the scleroderma model, the cells were stained with surface marker-specific following antibodies in the dark at 4°C for 30 mins: anti-mouse CD45 (clone 30-F11, BD Pharmingen), anti-mouse TCRβ (clone H57-597, BD Horizon), anti-mouse TCRγδ (clone GL3, BioLegend), anti-mouse CD4 (clone RM4-5, BD Horizon) and anti-mouse CD8α (clone 53-6.7, BioLegend), anti-human CCR6 (clone G034E3, BioLegend), anti-mouse Ly6G (clone 1A8, BioLegend), and anti-mouse CD11b (clone M1/70, BD Pharmingen). Dead cells were excluded using 7-AAD (BD Pharmingen).

For the psoriasis model, myeloid and T cell populations were analyzed using the following antibodies: anti-mouse CD45 (clone 30-F11, BD Pharmingen), anti-mouse TCRβ (clone H57-597, BD Horizon), anti-mouse TCRγδ (clone GL3, BioLegend), anti-mouse CD4 (clone RM4-5, BD Horizon), anti-mouse CD8α (clone 53-6.7, BioLegend), and anti-human CCR6 (clone G034E3, BioLegend). Dead cells were excluded using 7-AAD (BD Pharmingen).

In the CIA model, surface staining of myeloid and T cell populations was performed using the following antibodies: anti-mouse CD45 (clone 30-F11, BD Pharmingen), anti-mouse TCRβ (clone H57-597, BD Horizon), anti-mouse TCRγδ (clone GL3, BioLegend), anti-mouse CD4 (clone RM4-5, BD Horizon), anti-mouse CD8α (clone 53-6.7, BioLegend), anti-mouse CD19 (clone 1D3, BD Pharmingen), anti-mouse Ly-6G (clone 1A8, BioLegend), anti-mouse CD11b (clone M1/70, BioLegend), anti-mouse NK-1.1 (clone PK136, BD Horizon), and anti-mouse F4/80 (clone T45-2342, BD OptiBuild). Dead cells were excluded using the fixable viability stain 620 (BD Horizon).

For intracellular cytokine analyses, cells were stimulated *ex vivo* with 2 µL/mL cell stimulation cocktail (Tonbo Biosciences) for 4 hrs at 37 °C. Following stimulation, cells were washed with FACS buffer, fixed and permeabilized with BD fixation/permeabilization solution (BD Biosciences) in the dark at 4°C for 20 mins. Cells were then washed with FACS buffer and intracellularly stained with anti-mouse IL-17A (clone TC11-18H10.1, BioLegend) and anti-mouse IFN-γ (clone XMG1.2, BioLegend) in the dark at 4°C for 30 minutes. Dead cells were excluded using the fixable viability stain 620 (BD Horizon). Cell counts were determined using CountBright absolute counting beads (Life Technologies) following the manufacturer’s instructions. Flow cytometry data were acquired on a BD LSRFortessa X-20 (BD Biosciences) and analyzed using FlowJo software v10 (FlowJo, LLC).

### 2.11 Statistics

Data analysis and all graphical representations were performed using Prism 10.6.0 (GraphPad Software). Quantitative data were presented as mean ± standard error of the mean. Comparison between multiple groups was analyzed using two-way analysis of variance or one-way analysis of variance followed by Tukey’s multiple comparison test. Statistical significance was defined as a p-value of <0.05.

## 3 Results

### 3.1 αhCCR6 mAb attenuates skin inflammation and fibrosis by limiting pathogenic immune cell infiltration in a BLM-induced scleroderma model

**Figure 1:**
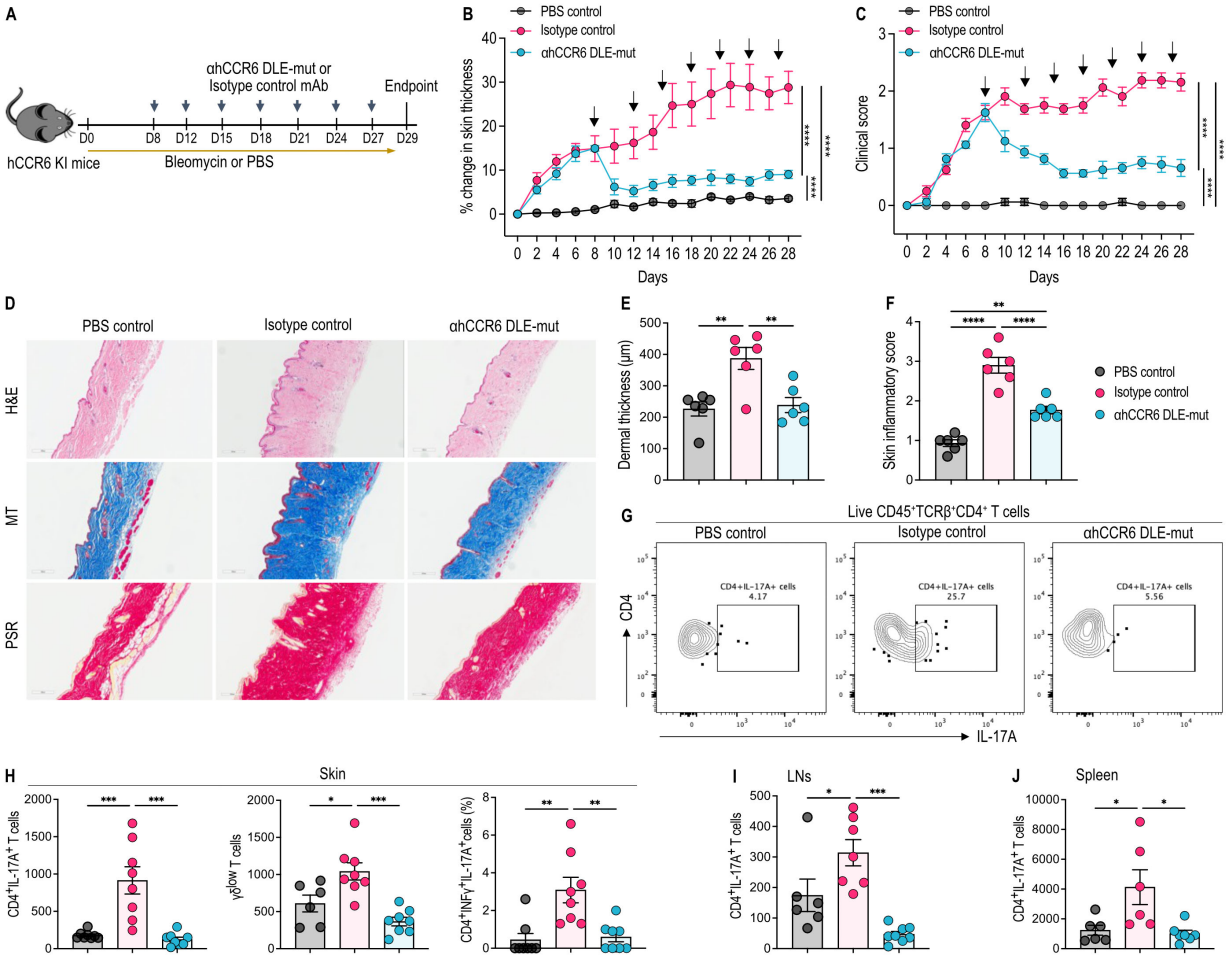
αhCCR6 mAb treatment attenuates BLM-induced skin inflammation and reduces pathogenic immune cell infiltration. hCCR6-Tg/mCCR6^-^/^-^ mice were subcutaneously injected with 100µg of Bleomycin (BLM) or PBS (as control) for 28 days. On day 8, when dorsal skin thickening (~15%) indicated disease progression and the onset of fibrotic signs (32, 33), mice were administered intraperitoneally (i.p.) a loading dose (25 mg/kg of body weight) of either an αhCCR6 mAb or an isotype control mAb, followed by maintenance doses (5 mg/kg, i.p.) twice a week. **(A)** Schematic of the treatment regimen **(B)** Dorsal skin thickness was measured on every other day, and the percent change was calculated compared with Day 0 baseline values. **(C)** Scleroderma clinical score was measured every other day. **(D)** Representative skin sections stained with H&E, Masson’s trichrome (MT), and Picrosirius red (PSR). Original magnification = ×20. **(E)** Dermal thickening was measured in tissue sections stained with H&E and quantified by ImageJ software (National Institutes of Health). **(F)** Skin sections stained with H&E were scored for the severity of the skin disease (as described in the Materials and Methods). Single-cell preparations were prepared from the skin, spleen and skin-draining LNs and analyzed by flow cytometry. **(G)** Representative flow plots of CD4^+^IL-17A^+^ T cells in the skin **(H)** the number of CD4^+^IL-17A^+^ T cells, γδ-low expressing T cells, and the percentage of Th17.1 (IFNγ^+^IL-17A^+^ CD4^+^) T cells in the skin, **(I)** the number of CD4^+^IL-17A^+^ T cells in the skin-draining LNs, **(J)** the number of CD4^+^IL-17A^+^ T cells in the spleen. All data represented as means ± SEM; n = 6 to 8 mice for each group. Statistics were calculated using **(B, C)** two-way analysis of variance and **(E, F, H–J)** one-way analysis of variance followed by Tukey’s multiple comparison test. *P < 0.05, **P < 0.01, ***P < 0.001, ****P < 0.0001.

To assess the therapeutic efficacy of Fc-enhanced αhCCR6 mAb in modulating fibrotic disease, we employed a bleomycin (BLM)-induced scleroderma model in human CCR6 knock-in (hCCR6 KI) mice. Subcutaneous BLM administration over four weeks induced hallmark features of scleroderma, including progressive dermal thickening, collagen deposition, and immune cell infiltration, closely mimicking the human disease phenotype (37). Treatment with αhCCR6 mAb was started on day 8, when displaying the onset of clinical symptoms (~15% increase in dorsal skin thickness and early fibrotic changes (32, 33) (**Figures 1A, B**).

*In vitro* characterization confirmed that our Fc-engineered αhCCR6 DLE-mut mAb exhibited potent, dose-dependent cytotoxicity against hCCR6^+^ target cells when co-incubated with human NK effector cells. Compared to the αhCCR6 Fc-Null (a CCR6-blocking antibody with a silenced Fc domain) and human IgG1 isotype control mAbs, αhCCR6 DLE-mut mAb significantly enhanced NK cell-mediated killing via ADCC mechanisms (**Supplementary Figure S1**).

Therapeutic administration of αhCCR6 DLE-mut mAb led to a marked reduction in dorsal skin thickness, a hallmark of skin inflammation and fibrosis, compared to isotype control-treated mice (**Figure 1B**). This was accompanied by a substantial decrease in cumulative clinical scores, which included assessments of skin thickening, erythema, and scaling (**Figure 1C**). Histopathological analysis of dorsal skin sections stained with H&E, MT, and PSR revealed that BLM injections markedly induced classic scleroderma features, such as dermal thickening, excessive collagen deposition, loss of subcutaneous adipose tissue replaced by fibrotic matrix, and extensive leukocyte infiltration (**Figures 1D–F**). Remarkably, these pathological changes were substantially alleviated in mice treated with Fc-enhanced αhCCR6 mAb, indicating the therapeutic potential of this approach in limiting fibrotic and inflammatory responses.

In scleroderma, various immune cell subsets infiltrate into the target organs during the early stages of systemic fibrosis development (14, 36, 38). To further investigate the immunomodulatory effects of antibody treatment, we analyzed immune cell infiltration into affected tissues, including inflamed skin, lung, skin-draining LNs, and the spleen, using flow cytometry. We observed that BLM injections markedly increased the numbers of various leukocyte populations in these sites, with a pronounced elevation of IL-17A-producing Th17 cells (CD4^+^IL-17A^+^ T cells) compared to PBS control (**Figures 1G–J**). Notably, αhCCR6 mAb treatment significantly reduced the number of pathogenic Th17 cell infiltrates compared to the isotype control (**Figures 1G–J**). BLM also elevated γδ-low expressing T cells in the inflamed skin, a primary source of IL-17 during inflammation (9), which were effectively reduced by Fc-enhanced αhCCR6 mAb treatment. (**Figure 1H**).

Additionally, BLM administration significantly increased the total number of CD45^+^ leukocytes in the skin, skin-draining LNs, and spleen (**Supplementary Figures S2A-C**). Our αhCCR6 mAb treatment led to a significant reduction in CD45^+^ cell numbers in the skin-draining LNs (**Supplementary Figure S2B**), whereas reductions in skin and spleen were not statistically significant (**Supplementary Figures S2A, C**). Importantly, αhCCR6 mAb treatment led to a significant decrease in CD4^+^ T cell numbers in the skin compared to isotype control, whereas other leukocyte subsets, including CD8^+^ T cells and neutrophils, remained unaffected (**Supplementary Figure S2A**). No significant differences were observed in the number of CD4^+^, CD8^+^, or γδ T cell populations in LNs or spleen (**Supplementary Figures S2B, C**).

Interestingly, BLM induced an increase in the frequency of CD4^+^IFNγ^+^IL-17A^+^ T cells (also known as Th17.1 cells) in the inflamed skin, whereas our αhCCR6 mAb significantly reduced this population compared to the isotype control (**Figure 1H**). Th17.1 cells represent a subset of Th17 cells that secrete both IL-17 and IFN-γ, thus playing a critical role in the pathogenesis of autoimmune diseases, including scleroderma (39–41).

These findings demonstrate that Fc-enhanced αhCCR6 mAb effectively limits fibrotic and inflammatory responses in the skin by selectively targeting CCR6^+^ pathogenic subsets, thereby attenuating scleroderma-associated inflammation.

### 3.2 αhCCR6 mAb inhibits lung inflammation and fibrosis through suppression of pathogenic immune cell recruitment in established BLM-induced scleroderma

Pulmonary fibrosis is a major complication in patients with scleroderma, and a leading cause of mortality in this population (42, 43). The BLM-induced scleroderma model also recapitulates key features of bronchoalveolar inflammation observed in humans, including lung fibrosis, alveolar damage, collagen deposition, and immune cell infiltration (36, 44).

**Figure 2:**
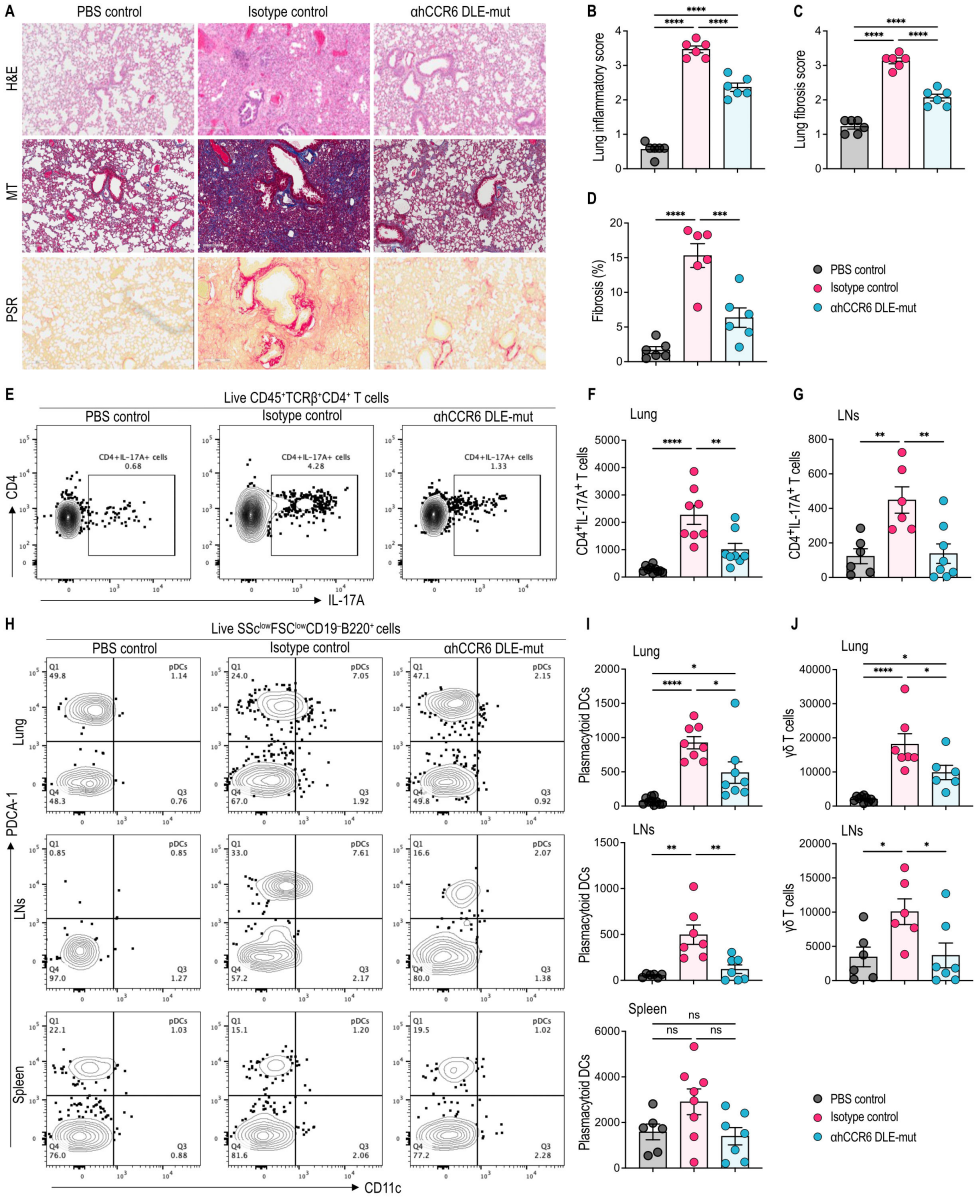
αhCCR6 mAb treatment reduces BLM-induced lung inflammation, fibrosis and leukocyte infiltration. hCCR6-Tg/mCCR6^-/-^ mice were subcutaneously injected with 100 µg of Bleomycin (BLM) or PBS (as control) for 28 days. On day 8, when dorsal skin thickening (~15%) indicated disease progression and the onset of fibrotic signs (32, 33), mice were administered intraperitoneally (i.p.) a loading dose (25 mg/kg of body weight) of either an αhCCR6 mAb or an isotype control mAb, followed by maintenance doses (5 mg/kg, i.p.) twice a week. **(A)** Representative lung tissue sections stained with H&E, Masson’s trichrome (MT), and Picrosirius red (PSR). Original magnification = ×20, **(B)** Lung tissue sections stained with H&E were scored for the severity of lung disease (as described in the Materials and Methods). **(C, D)** Lung tissue sections stained with MT were scored for fibrosis as the modified Ashcroft score (35, 36) (as described in Materials and Methods), and the percentage of lung fibrosis was quantified by ImageJ software (National Institutes of Health). Single-cell preparations were prepared from the lung, lung-draining LNs and spleen, and analyzed by flow cytometry. **(E)** Representative flowplots of CD4^+^IL-17A^+^ T cells in the lung. **(F, G)** the number of CD4^+^IL-17A^+^ T cells in the lung and lung-draining LNs. **(H)** Representative flow plots identifying plasmacytoid dendritic cells (pDCs) as live SSC^low^FSC^low^CD19^-^B220^+^ CD11c^int^PDCA-1^+^ cells within the lymphocyte gate. **(I)** the number of pDCs infiltration in the lung, lung-draining LNs, and spleen. **(J)** the number of γδ T cells in the lung and lung-draining LNs. All data represented as means ± SEM; n = 6 to 8 mice for each group. Statistics were calculated using one-way analysis of variance followed by Tukey’s multiple comparison test. *P < 0.05, **P < 0.01, ***P < 0.001, ****P < 0.0001.

Histological examination of lung tissue sections stained with H&E, MT, and PSR revealed severe pathological alterations in BLM-treated mice, including disruption of normal alveolar architecture, dense collagen deposition, and extensive leukocyte infiltration, compared to PBS control animals (**Figure 2A**). Treatment with the Fc-enhanced αhCCR6 mAb substantially reduced these pathological features, as reflected by lower lung inflammation and fibrosis scores compared with animals receiving an isotype control mAb (**Figures 2B–D**).

Flow cytometric analysis showed that BLM injections markedly increased the number of CD4^+^IL-17A^+^ Th17 cells and γδ T cells in the lungs and lung-draining LNs of mice receiving isotype control mAb compared with PBS controls. Remarkably, αhCCR6 mAb treatment significantly decreased the abundance of these pathogenic immune subsets in these tissues (**Figures 2E–G**, and **J**). Similarly, BLM injections induced substantial infiltration of plasmacytoid dendritic cells (pDCs) into both the lung and lung-draining LNs, which was markedly reduced following αhCCR6 mAb treatment (**Figures 2H, I**). These cells play a crucial role in the development of lung fibrosis by accumulating in the lungs and facilitating the recruitment of pathogenic immune cells to the affected tissues (36). However, their numbers remained unchanged in the spleen following BLM injections, indicating tissue-specific migration in response to BLM-induced inflammation (**Figures 2H, I**).

Although BLM injections also elevated total CD45^+^ leukocytes, CD4^+^, and CD8^+^ T cells in the lung, αhCCR6 mAb treatment did not significantly alter these populations (**Supplementary Figure S2D**). However, CD11c^+^CD11b^+^ myeloid dendritic cells (DCs) were elevated in both the lung and lung-draining LNs following BLM exposure, and αhCCR6 treatment selectively reduced their numbers in the LNs, while their numbers remained elevated in lung tissue (**Supplementary Figures S2D, E**). Similarly, total CD45^+^ leukocyte numbers were reduced in the LNs following αhCCR6 treatment but remained unchanged in lung tissue, while CD4^+^ and CD8^+^ T cells were unaffected at both sites (**Supplementary Figures S2D, E**).

Together, these results demonstrate that αhCCR6 mAb treatment suppresses lung inflammation and fibrosis by suppressing the recruitment of pathogenic Th17 cells, γδ T cells, and pDCs, supporting the CCR6-targeted approach as a promising therapeutic strategy for pulmonary complications in scleroderma.

### 3.3 αhCCR6 mAb suppresses skin inflammation and reduces pathogenic immune cell infiltration in the IMQ-induced psoriasis model

**Figure 3:**
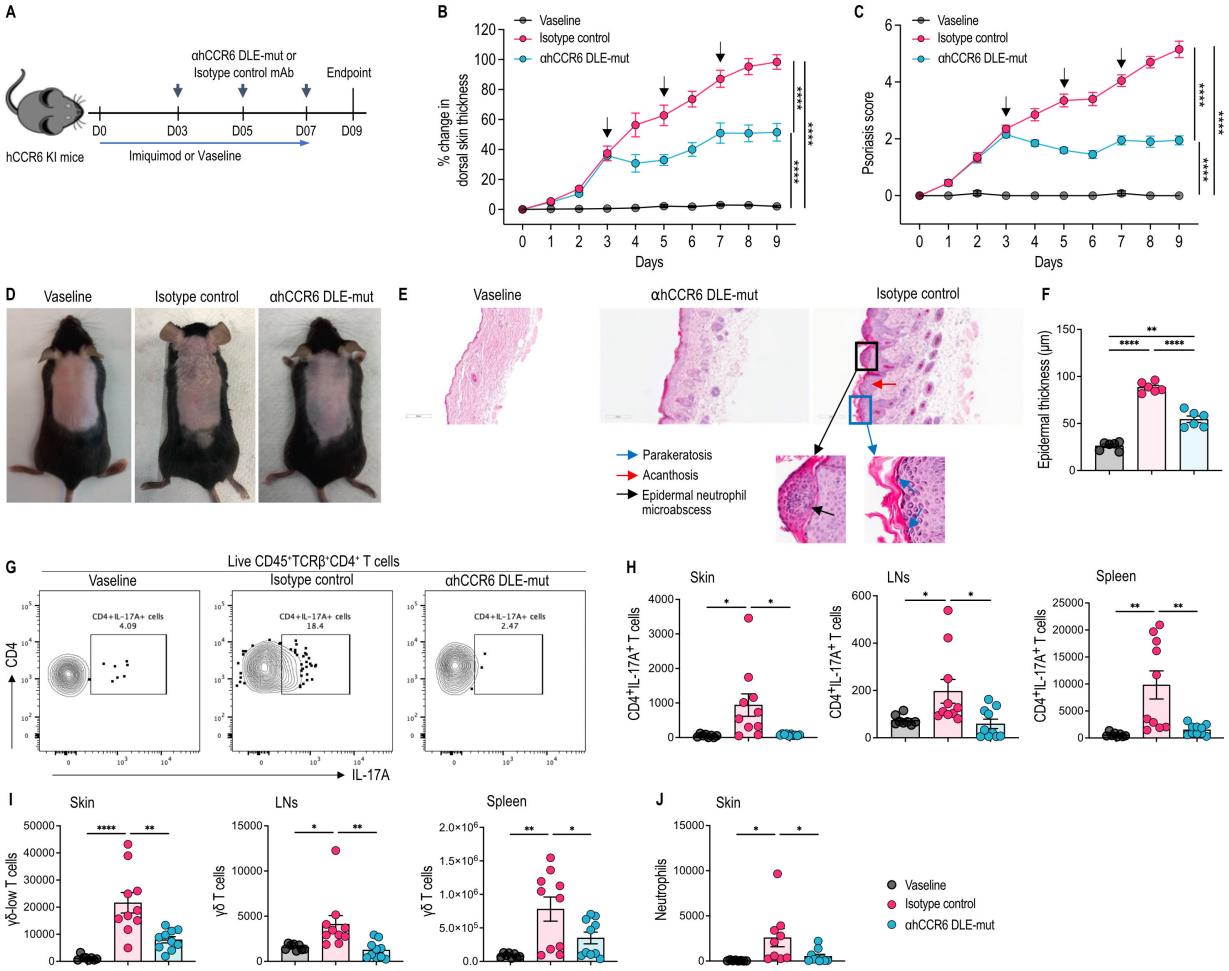
αhCCR6 mAb inhibits skin inflammation and prevents leukocyte infiltration in established IMQ-induced psoriasis. hCCR6-Tg/mCCR6^-/-^ mice were topically applied with 20 mg imiquimod (IMQ) 5% cream or Vaseline (as control) for 7 consecutive days. On day 3, when dorsal skin thickening (~30%) exhibited symptoms of the disease (13), mice were administered intraperitoneally (i.p.) a loading dose (25 mg/kg of body weight) of either an αhCCR6 mAb or an isotype control, followed by maintenance doses (5 mg/kg, i.p.) every other day for a week. **(A)** Schematic of the treatment regimen **(B)** Dorsal skin thickness was measured on each treatment day and the percent change was calculated compared with Day 0 baseline values **(C)** Psoriasis score was measured on each treatment day (as described in the Materials and Methods) **(D)** Representative appearance of dorsal skin at the endpoint **(E)** Representative skin sections stained with H&E. Original magnification = ×20. **(F)** Epidermal thickening was measured in tissue sections stained with H&E and quantified by ImageJ software (National Institutes of Health). Single-cell preparations from dorsal skin, skin-draining lymph nodes and spleen were prepared and analyzed by flow cytometry to assess the effects of αhCCR6 mAb treatment on immune cell infiltration. **(G)** Representative flowplots of CD4^+^IL-17A^+^ T cells in the skin. **(H)** the number of CD4^+^IL-17A^+^ T cells in the skin, skin-draining LNs and spleen. **(I)** the number of γδ T cells in the skin, skin-draining LNs and spleen. **(J)** the number of neutrophils in the skin. All data represented as means ± SEM; n = 8 to 10 mice for each group. Statistics were calculated using **(B, C)** two-way analysis of variance and **(F, H–J)** one-way analysis of variance followed by Tukey’s multiple comparison test. *P < 0.05, **P < 0.01, ****P < 0.0001.

Topical application of IMQ cream induces psoriatic skin inflammation in mice similar to human plaque-type psoriasis (45). To examine whether targeting CCR6^+^ cells alters disease severity, we administered Fc-enhanced anti-CCR6 mAb to IMQ-treated hCCR6 KI mice, which exhibited evident signs of the disease (~30% increase in dorsal skin thickening) on day 3 (**Figures 3A, B**).

Consistent with our previous findings using a CCR6-blocking mAb (13), treatment with the Fc-enhanced αhCCR6 mAb significantly alleviated disease symptoms in mice with established disease. This therapeutic benefit was evident through a substantial reduction in dorsal skin thickening, which serves as a surrogate marker of skin inflammation, as well as decreased skin redness and scaling compared to isotype control-treated animals (**Figures 3B–D**). Histopathological analysis further revealed that Fc-enhanced anti-CCR6 mAb exhibited marked suppression of hallmark features of psoriatic pathology, including acanthosis (epidermal thickening), parakeratosis, neutrophil microabscess formation, and dermal inflammatory infiltrates, compared to mice treated with an isotype control mAb (**Figures 3E, F**).

Additionally, Fc-enhanced anti-CCR6 mAb treatment significantly reduced the infiltration of inflammatory cells into the inflamed skin, including CD4^+^IL-17A^+^ Th17 cells, and γδ-low expressing T cells, compared to mice treated with IMQ and receiving the isotype control mAb (**Figures 3G–I**). IMQ exposure also increased Th17 and γδ T cells infiltration into the skin-draining LNs and spleen (**Figures 3H, I**). Th17 cells, which express high levels of CCR6 (1), play a central role in the pathogenesis of this disease (8, 13, 45, 46). In addition, γδ-low T cells are a distinct dermal immune cell population (9) that serves as a major source of IL-17 during psoriatic skin inflammation and also express elevated levels of CCR6 (8, 10, 11). Notably, Fc-enhanced anti-CCR6 mAb treatment significantly reduced Th17 and γδ T cell numbers in both the skin-draining LNs and spleen (**Figures 3H, I**). Likewise, neutrophil infiltration, a key driver of psoriatic pathology (47), was also significantly diminished in the skin following αhCCR6 treatment (**Figure 3J**).

Analysis of broader immune populations showed that the antibody treatment decreased total CD45^+^ leukocytes and CD4^+^ T cells in the skin, whereas CD8^+^ T cells and dendritic epidermal T cells (DETCs) remained unaffected (**Supplementary Figure S3A**). In contrast, CCR6 targeting did not significantly affect the infiltration of these immune cell populations in the skin-draining LNs or spleen (**Supplementary Figure S3B, C**).

These findings demonstrate that targeting of CCR6^+^ cells with αhCCR6 mAb effectively suppresses psoriatic inflammation by preventing infiltration of pathogenic Th17 cells, γδ T cells, and neutrophils into inflamed tissues, underscoring the therapeutic promise of CCR6-targeted strategies for psoriasis and other IL-17/IL-22–driven diseases.

### 3.4 αhCCR6 mAb treatment alleviates disease severity in the collagen induced arthritis model

**Figure 4:**
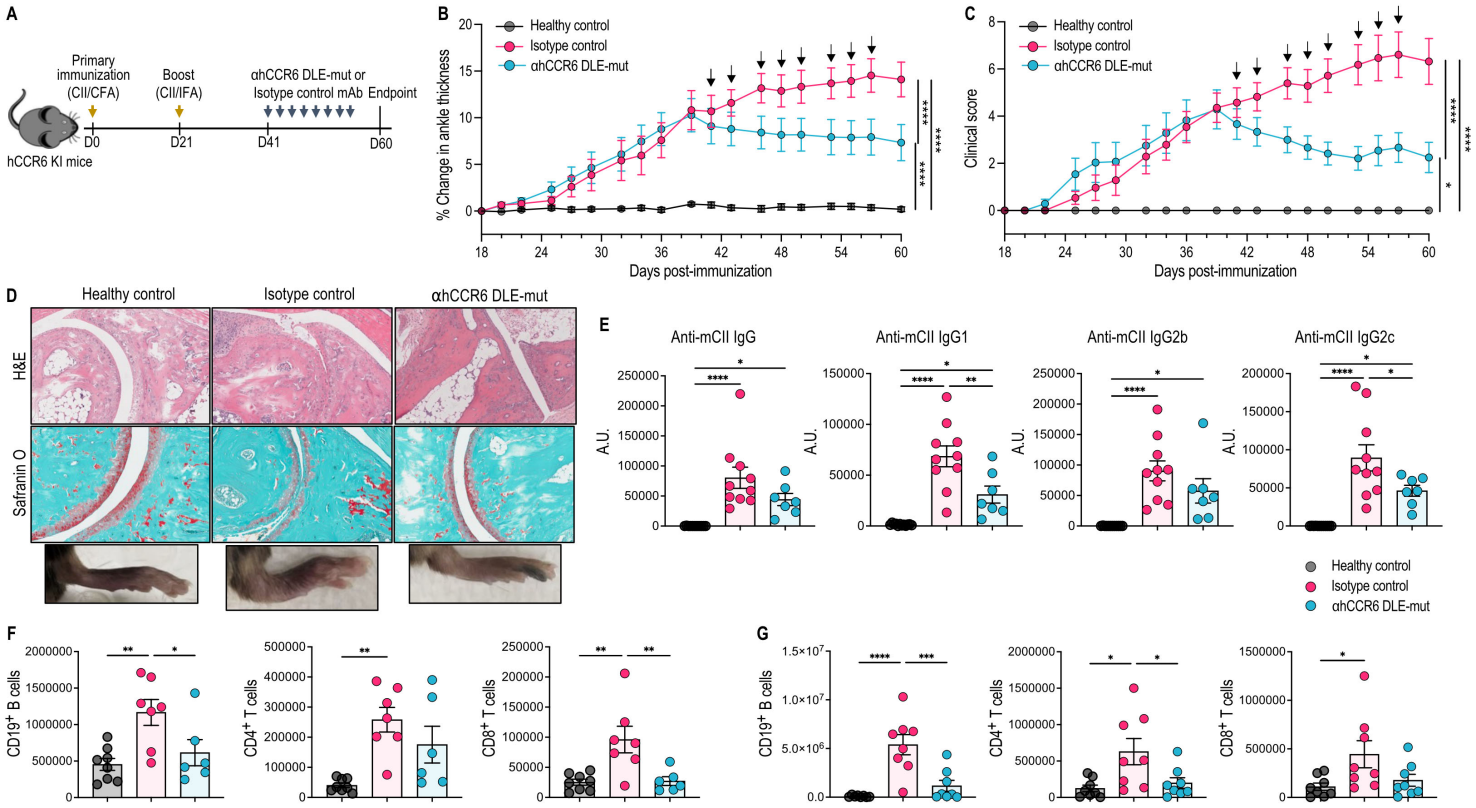
αhCCR6 mAb reduces joint inflammation, immune cell infiltration, and anti-collagen autoantibodies in collagen-induced arthritis (CIA). hCCR6-Tg/mCCR6^-^/^-^ mice were subcutaneously injected with an emulsion of 100 µg type II collagen (anti-CII) and 200 µg *M. tuberculosis* in Complete Freund’s Adjuvant (CFA) on Day 0 and given a booster injection of CII emulsified with Incomplete Freund’s adjuvant (IFA) on Day 21 to induce CIA. When mice exhibited swelling in ankle joints and front paws with an average cumulative clinical score of 4 (day 41), mice were treated with intraperitoneal (i.p.) injections of 5 mg/kg of body weight with αhCCR6 or isotype control mAbs, every other day for 3 weeks. On day 60, mice were humanely killed, and blood, ankle joints and popliteal LNs were collected for the measurement of CII-specific autoantibodies, cellular infiltrates, and histological analysis. **(A)** Schematic of the treatment regimen. **(B)** Measurement of ankle thickness of healthy control (n=12), Isotype control (n=14), and αhCCR6 mAb (n=14) groups and **(C)** clinical arthritis scoring. **(D)** Representative ankle joint sections stained with H&E and Safranin O. **(E)** Measurement of anti-CII specific antibodies in the serum. Analysis of the number of immune cells in: **(F)** ankle joints and **(G)** popliteal LNs. All data represented as means ± SEM; n = 6 to 8 mice for each group. Statistics were calculated using **(B, C)** two-way analysis of variance and **(E–G)** one-way analysis of variance followed by Tukey’s multiple comparison test. *P < 0.05, **P < 0.01, ***P < 0.001, ****P < 0.0001.

We further explored the therapeutic potential of targeting CCR6 using Fc-enhanced anti-CCR6 mAb treatment in another model of autoimmunity: the CIA model, a well-established model of autoimmune arthritis. In a therapeutic setting, the anti-CCR6 mAb was administered to hCCR6 KI mice with clinically evident CIA (average cumulative clinical score of 4 on day 41, characterized by swelling in the ankle joints and forepaws; **Figures 4A–C**). Our results showed that αhCCR6 mAb treatment significantly alleviated clinical symptoms of arthritis, as evidenced by reduced ankle thickening (a surrogate indicator of arthritis) and lower clinical scores, compared to the isotype control-treated animals (**Figures 4B, C**).

Histopathological assessment of the ankle joints confirmed these clinical improvements by displaying a notable reduction in inflammatory cell infiltration (as shown in H&E-stained sections) and preservation of normal joint structure, with minimal loss of cartilage proteoglycan at articular surfaces (as shown in Safranin O-stained sections), in the Fc-enhanced anti-CCR6 mAb treated animals compared to the isotype control group (**Figure 4D**).

Considering the pathogenic role of anti-collagen type II (CII) antibodies in CIA, we measured circulating levels of anti-CII antibodies. These anti-CII-specific Abs are known to be arthritogenic and are essential for the development of CIA (48–50). We observed that αhCCR6 mAb treatment significantly reduced anti-CII-specific Ab titers, including anti-CII IgG1 and anti-CII IgG2c titers, compared to the isotype control, indicating attenuation of the pathogenic humoral response (**Figure 4E**).

Immune profiling of inflamed joints and popliteal draining LNs showed that αhCCR6 mAb significantly reduced the number of CD19^+^ B cells and CD8^+^ T cells in the joints, while CD4^+^ T cells remained unchanged (**Figure 4F**). In the draining LNs, αhCCR6 mAb reduced CD19^+^ B cells and CD4^+^ T cells, but not CD8^+^ T cells, when compared to mice treated with isotype control mAb (**Figure 4G**). Total CD45^+^ leukocytes and γδ T cells numbers in the joints were unaffected, and although Th17 cell numbers trended lower, the reduction was not statistically significant (**Supplementary Figure S4A**). Infiltration of innate immune cells, including neutrophils, NK cells, and F4/80^+^ macrophages, was also unchanged in the joints (**Supplementary Figure S4A**). However, in draining LNs, CCR6 targeting significantly reduced CD45^+^ leukocytes and γδ T cells, but not Th17 cells (**Supplementary Figure S4B**).

Collectively, these results demonstrate that targeting CCR6^+^ cells using Fc-enhanced anti-CCR6 mAb treatment effectively alleviates CIA by dampening pathogenic anti-CII Abs responses and limiting infiltration of disease-associated immune cells into affected tissues. These findings underscore the role of CCR6^+^ immune cells in arthritis pathogenesis and support therapeutically targeting CCR6^+^ cells as a promising strategy for IL-17-driven autoimmune diseases.

## 4 Discussion

The migration of leukocytes to the site of inflammation is essential to innate immunity for host defense and maintaining immune homeostasis. Nevertheless, uncontrolled infiltration and activation of leukocytes into tissues can drive chronic inflammatory responses, leading to various pathologies, including autoimmune diseases. Among the key regulators of immune cell trafficking is the chemokine receptor CCR6, which is highly expressed on IL-17-producing Th17 cells and other pathogenic immune subsets and facilitates their directed migration to inflamed tissues, thus amplifying inflammatory responses and disease progression (1, 5–7). Consequently, antagonism or targeted depletion of this receptor holds considerable promise as an anti-inflammatory therapeutic strategy in inflammatory and autoimmune diseases. Numerous studies using CCR6-deficient mice or pharmacological inhibitors have demonstrated the critical role of this receptor in autoimmune and inflammatory disorders, leading to diminished inflammation in numerous animal models (1, 10, 26–28). Despite these therapeuticpromises, no effective mAb against CCR6 has advanced to clinical studies (51), and only one small molecule inhibitor (PF-07054894) is currently undergoing phase 1 clinical trials (52). To address this gap, we have previously developed a fully humanized mAb against hCCR6 with potent antagonistic activity, which has shown efficacy in preclinical models of EAE and psoriasis (13).

Targeting CCR6 with mAb offers distinct advantages over neutralizing its ligand CCL20 due to the high levels of circulating CCL20, which can limit the efficacy of ligand-blocking strategies. Moreover, prior attempts to develop antibodies against CCL20 were halted after a Phase 1 trial due to immune complex–mediated toxicity observed in non-human primates (53). Furthermore, CCR6 binds to non-chemokine ligands, such as human β-defensin-1 and 2, which are upregulated during inflammation and contribute to the recruitment of Th17 cells to inflammation sites (54–56). These factors underscore the therapeutic rationale for directly targeting CCR6 rather than its ligands for treating inflammatory and autoimmune conditions. However, antagonizing chemokine receptors as a therapeutic approach has been quite disappointing, and only two small molecules against CXCR4 and CCR5 have been approved to date for human use.

In this study, we investigated the therapeutic efficacy of a Fc-enhanced anti-CCR6 mAb across multiple animal models of autoimmunity: BLM-induced scleroderma, IMQ-induced psoriasis, and CIA. In the scleroderma model, therapeutic administration of αhCCR6 mAb resulted in a substantial reduction of skin inflammation and fibrosis, accompanied by a marked decrease in pathogenic immune cells, specifically IL-17-producing Th17 cells and Th17.1 cells, in the skin and skin-draining LNs. This observation following αhCCR6 mAb treatment highlights the critical role of CCR6^+^ cells in mediating inflammation, as well as the antibody’s capacity to target highly pathogenic immune cell subsets. Studies in animal models and scleroderma patients have shown that Th17 cells contribute to the pathogenesis of this disease through the production of IL-17 (14, 21, 38, 57). Furthermore, Th17 cells secrete a spectrum of pro-inflammatory cytokines, including IFN-γ, IL-21 and IL-22 (58, 59). In alignment with this, we observed an increased frequency of IFN-γ-producing Th17.1 cells in the inflamed skin following BLM administration, and treatment with our Fc-enhanced anti-CCR6 mAb significantly diminished these inflammatory infiltrates. Th17.1 cells, a subset of Th17 cells, secrete both IL-17 and IFN-γ cytokines, thereby contributing critically to the pathogenesis of autoimmune diseases, including scleroderma (39–41). Previous studies have documented high levels of IL-17 and IFN-γ in the serum of scleroderma patients (39). A recent study reported a significant increase in the percentage of these Th17 cell subsets in the blood of scleroderma patients, facilitating fibroblast proliferation and collagen synthesis, which subsequently contributes to the dermal fibrosis observed in this condition (41). Additionally, we observed that selective targeting of CCR6 using the αhCCR6 mAb resulted in a marked reduction of γδ-low T cells within the skin and skin-draining LNs. These cells exhibit higher levels of CCR6 (8) and are a major source of IL-17 (9, 12) during inflammation. These pathogenic subsets of γδ T cells are known to migrate to inflamed tissues and contribute to disease progression through the production of high levels of Th17 cytokines (10, 12, 13).

Beyond cutaneous manifestations, BLM also induces lung inflammation and fibrosis (36, 44). In humans, pulmonary fibrosis is one of the main complications of scleroderma and is the leading cause of death among affected patients (42, 43). Remarkably, our αhCCR6 mAb significantly attenuated lung inflammation and fibrosis in the BLM-induced scleroderma model. This therapeutic benefit was associated with reduced infiltration of Th17 cells, γδ T cells, and pDCs in both the lungs and lung-draining LNs. These cell types produce IL-17A (14, 38), TNF-α (60), and other pro-fibrotic mediators that drive lung inflammation and fibrosis (61, 62).

Interestingly, BLM injections selectively increase pDCs infiltration to the lungs and lung-draining LNs, but not the spleen, indicating targeted migration to affected tissues. These cells drive lung fibrosis by recruiting pathogenic immune cells and promoting pro-inflammatory and pro-fibrotic responses. Their reduction following mAb treatment reduces fibrosis and inflammatory infiltrates in both the skin and lungs (36). In scleroderma patients, elevated levels of pDCs in the lungs are associated with disease severity (36). pDCs infiltrate the lungs (63) and are potent producers of CXCL4 and IFN‐α, both of which are implicated in fibrotic responses and disease severity (64, 65).

While γδ T cells are another key producer of IL-17A and known contributors to IL-17–mediated inflammation (66), their role in fibrosis is context-dependent. In some settings, their depletion may exacerbate disease, suggesting a dual role in immune regulation (67). Nonetheless, our data support their pathogenic contribution in the BLM model and highlight the therapeutic value of CCR6^+^ cell targeting.

In the psoriasis model, our Fc-enhanced anti-CCR6 mAb demonstrated robust therapeutic efficacy in suppressing psoriatic skin inflammation. Building on our previous work of antagonizing CCR6 using a CCR6-blocking mAb in a prophylactic setting of a preclinical model (13), this study evaluated the therapeutic potential of CCR6^+^ cell targeting with Fc-enhanced mAb after disease onset, which more closely reflects clinical treatment scenarios. Notably, the αhCCR6 mAb used here was not identical to that used in our earlier study: the current mAb was Fc-engineered to enhance binding affinity to both human and murine Fcγ receptors, thereby increasing ADCC and ADCP and enabling selective depletion of CCR6^+^ immune cells. By repeating the psoriasis experiment with this Fc-enhanced anti-CCR6 mAb, we directly assessed whether this new CCR6-targeted approach retains therapeutic potency under clinically relevant conditions. Thus, while our previous blocking antibody primarily interfered with CCR6–CCL20–driven migration, the Fc-enhanced αhCCR6 DLE-mut mAb additionally recruits FcγR-bearing effector cells and therefore has the potential to exert deeper and more sustained control of IL-17–driven inflammation than receptor blockade alone.

Treatment with αhCCR6 mAb demonstrated robust therapeutic benefits and significantly reduced hallmark features of psoriasis, such as skin thickening, erythema, and scaling. It also markedly decreased the infiltration of IL-17A-producing Th17 cells and γδ-low T cells, key drivers of psoriatic pathology. These dermal γδ T cells, which express high levels of CCR6, are the predominant source of IL-17 in murine psoriasis models and have human counterparts implicated in psoriatic lesions (8, 10, 11). In humans, an equivalent dermal γδ T cell plays a similarly pathogenic role in psoriatic lesions (8). Our findings reinforce the therapeutic relevance of CCR6^+^ cell targeting and align with prior studies showing that genetic deletion or antibody-mediated blockade of CCR6 (13) or its ligand CCL20 (10) impairs disease development. These results validate αhCCR6 mAb as a promising candidate for psoriasis therapy and support broader application of CCR6-targeted strategies in other IL-17/IL-22–driven diseases.

In the CIA model, treatment with Fc-enhanced anti-CCR6 mAb effectively alleviated all clinical signs of arthritis, including reduced ankle swelling and lower cumulative disease scores. This therapeutic effect was accompanied by a reduction in circulating anti-CII IgG Abs and decreased infiltration of CD4^+^ and CD8^+^ T cells, as well as CD19^+^ B cells, into inflamed joints and draining LNs. These immune cell populations are central to RA pathogenesis (68–72) and frequently detected in the synovial fluid of RA patients (73–75). B cells, in particular, drive antigen-specific immune responses and contribute to disease progression through the production of rheumatoid factors and high-affinity autoantibodies (73). Their deficiency or depletion using mAb treatment has been shown to prevent arthritis onset in murine models (76, 77). Furthermore, CII-specific antibodies are known to exacerbate joint inflammation and are key contributors to CIA (49, 78), highlighting the coordinated role of cellular and humoral immune responses in disease progression (79).

The observed reduction in anti-CII Ab titers following αhCCR6 mAb treatment suggests that CCR6-targeting may modulate the T–B cell axis, as reflected by reduced anti-CII antibody titers and diminished B-cell numbers in the joints and draining lymph nodes, although we did not directly enumerate CCR6^+^ B, Tfh or Treg cell populations in this study. CCR6 expression is transiently upregulated on activated B cells and influences germinal center dynamics, thereby impacting antibody production (4, 80). Moreover, CCR6^+^ Th17 and Th17.1 cells are known to provide potent B cell help via secretion of IL-21 and other cytokines (81, 82). By targeting CCR6^+^ cells with a Fc-enhanced anti-CCR6 mAb, we most likely disrupt this T-B cell axis, which in turn attenuates both cellular and humoral drivers of arthritis progression.

While our findings demonstrate the broad therapeutic potential of CCR6^+^ cell targeting with an Fc-enhanced anti-CCR6 mAb across multiple pre-established autoimmune disease models, a few limitations should be considered. First, our analyses focused primarily on Th17 cells, γδ T cells, and pDCs, which are key drivers of autoimmune responses. However, we did not assess other CCR6^+^ populations, including B cells, innate lymphoid cells (ILCs), and plasma cells. In the CIA model, although we observed reduced anti-CII antibody levels due to the reduction of CCR6^+^ cells via Fc-enhanced mAb treatment, suggesting modulation of autoreactive B cell responses, a direct assessment of CCR6 expression on plasma cells is needed to confirm their involvement. Additionally, cytokine profiling in this study was limited to IL-17A and IFN-γ, and future studies should include other relevant mediators such as IL-22, TNF-α, and CCL20 to fully elucidate the immunological landscape. In addition, we did not perform dedicated pharmacokinetic and receptor occupancy in these models, and future translational work will need to define the relationships between exposure, target engagement, and modulation of CCR6-associated immune compartments.

Our findings demonstrate that therapeutic targeting of CCR6-expressing immune cells with an Fc-enhanced anti-CCR6 mAb is highly effective at reducing IL-17–driven inflammation across three pre-established autoimmune models. A limitation of our study is the lack of direct *in vivo* quantification of CCR6^+^ cells following antibody treatment. Indeed, the therapeutic antibody (clone 6H12) blocks the epitope recognized by available anti-CCR6 detection antibodies, precluding reliable staining of CCR6 after *in vivo* dosing. As a result, we infer engagement of CCR6^+^ targets indirectly from (i) robust NK-cell–mediated cytotoxicity *in vitro*, (ii) consistent reductions in IL-17–producing Th17 cells, γδ-17 cells, pDCs, and B cells in inflamed tissues, and (iii) preservation of CCR6-negative leukocyte subsets. Altogether, these results highlight CCR6 as a central regulator of pathogenic immune cell trafficking and function, supporting the development of CCR6-targeted interventions for IL-17-driven autoimmune and inflammatory pathologies where current therapies remain inadequate.

## Data Availability

The raw data supporting the conclusions of this article will be made available by the authors, without undue reservation.

## Supplementary material

Supplementary Figure 1 Functional characterization of αhCCR6 mAb in NK cell-mediated cytotoxicity assay. Activated human natural killer (NK) cells (effector) were co-cultured with hCCR6-expressing L1.2 target cells at a fixed effector-to-target (E:T) ratio of 4:1. Cytotoxic activity was assessed *in vitro* using increasing concentrations of αhCCR6 DLE-mut, αhCCR6 Fc-Null, or human IgG1 isotype control mAbs. A dose-dependent enhancement of NK cell-mediated killing was observed with αhCCR6 DLE-mut mAb, demonstrating its functional potency and target specificity.

Supplementary Figure 2 Effect of αhCCR6 mAb on immune cell populations in the skin, lung, draining lymph nodes, and spleen of bleomycin (BLM)-treated mice. hCCR6-Tg/mCCR6^-/-^ mice were subcutaneously injected with 100µg of Bleomycin or PBS (as control) for 28 days. On day 8, when dorsal skin thickening (~15%) indicated disease progression, and the onset of fibrotic signs (32, 33), mice were administered intraperitoneally (i.p.) a loading dose (25 mg/kg of body weight) of either αhCCR6 or an isotype control mAbs, followed by maintenance doses (5 mg/kg, i.p.) twice a week. At the end of the experiment, single-cell preparations were prepared from the dorsal skin, spleen and skin-draining LNs and analyzed by flow cytometry. The effect of αhCCR6 mAb treatment on the number of leukocyte subsets infiltration was assessed in: **(A)** Dorsal skin, **(B)** skin-draining LNs, **(C)** Spleen, **(D)** Lung, and **(E)** Lung-draining LNs. All data represented as means ± SEM; n = 6 to 8 mice for each group. Statistics were calculated using one-way analysis of variance followed by Tukey’s multiple comparison test. *P < 0.05, **P < 0.01, ***P < 0.001, ****P < 0.0001.

Supplementary Figure 3 Effect of αhCCR6 mAb on immune cell subsets in the skin, skin-draining lymph nodes, and spleen of imiquimod (IMQ)-treated mice. hCCR6-Tg/mCCR6^-/-^ mice were topically applied with 20 mg imiquimod (IMQ) 5% cream or Vaseline (as control) for 7 consecutive days. On day 3, when dorsal skin thickening (~30%) indicated disease onset, mice were administered intraperitoneally (i.p.) a loading dose (25 mg/kg of body weight) of either αhCCR6 or an isotype control mAbs, followed by maintenance doses (5 mg/kg, i.p.) every other day for a week. At the end of the experiment, single-cell preparations were obtained from the dorsal skin, skin-draining LNs, and spleen and analyzed by flow cytometry. The effect of αhCCR6 mAb treatment on the number of leukocyte subsets infiltration were assessed in: **(A)** Dorsal skin, **(B)** skin-draining LNs, and **(C)** Spleen. All data represented as means ± SEM; n = 8 to 10 mice for each group. Statistics were calculated using one-way analysis of variance followed by Tukey’s multiple comparison test. *P < 0.05, **P < 0.01, ***P < 0.001, ****P < 0.0001.

Supplementary Figure 4 Effect of αhCCR6 mAb on immune cell populations in the ankle and draining nodes in collagen-induced arthritis (CIA). hCCR6-Tg/mCCR6^-^/^-^ mice were subcutaneously injected with an emulsion of 100 µg type II collagen (anti-CII) and 200 µg *M. tuberculosis* in Complete Freund’s Adjuvant (CFA) on Day 0 and given a booster injection of CII emulsified with Incomplete Freund’s adjuvant (IFA) on Day 21 to induce CIA. When mice exhibited swelling in ankle joints and front paws with an average cumulative clinical score of 4 (day 41), mice were treated with intraperitoneal (i.p.) injections of 5 mg/kg of body weight with αhCCR6 or isotype control mAbs, every other day for 3 weeks. At the end of the experiment, single-cell preparations from the ankle and draining popliteal LNs were prepared and analyzed by flow cytometry. The effects of αhCCR6 mAb treatment on the number of leukocyte infiltrations were assessed in: **(A)** Ankle and **(B)** Draining popliteal LNs. All data represented as means ± SEM; n = 6 to 8 mice for each group. Statistics were calculated using one-way analysis of variance followed by Tukey’s multiple comparison test. *P < 0.05, **P < 0.01, ***P < 0.001, ****P < 0.0001.
