# Supplementary figures and images for: Fc-enhanced anti-CCR6 antibody elicits robust therapeutic effects across multiple autoimmune diseases
Source: Front Immunol. 2026 Jan 9;16:1728419. doi: 10.3389/fimmu.2025.1728419 (PMC12847933; doi:10.3389/fimmu.2025.1728419)

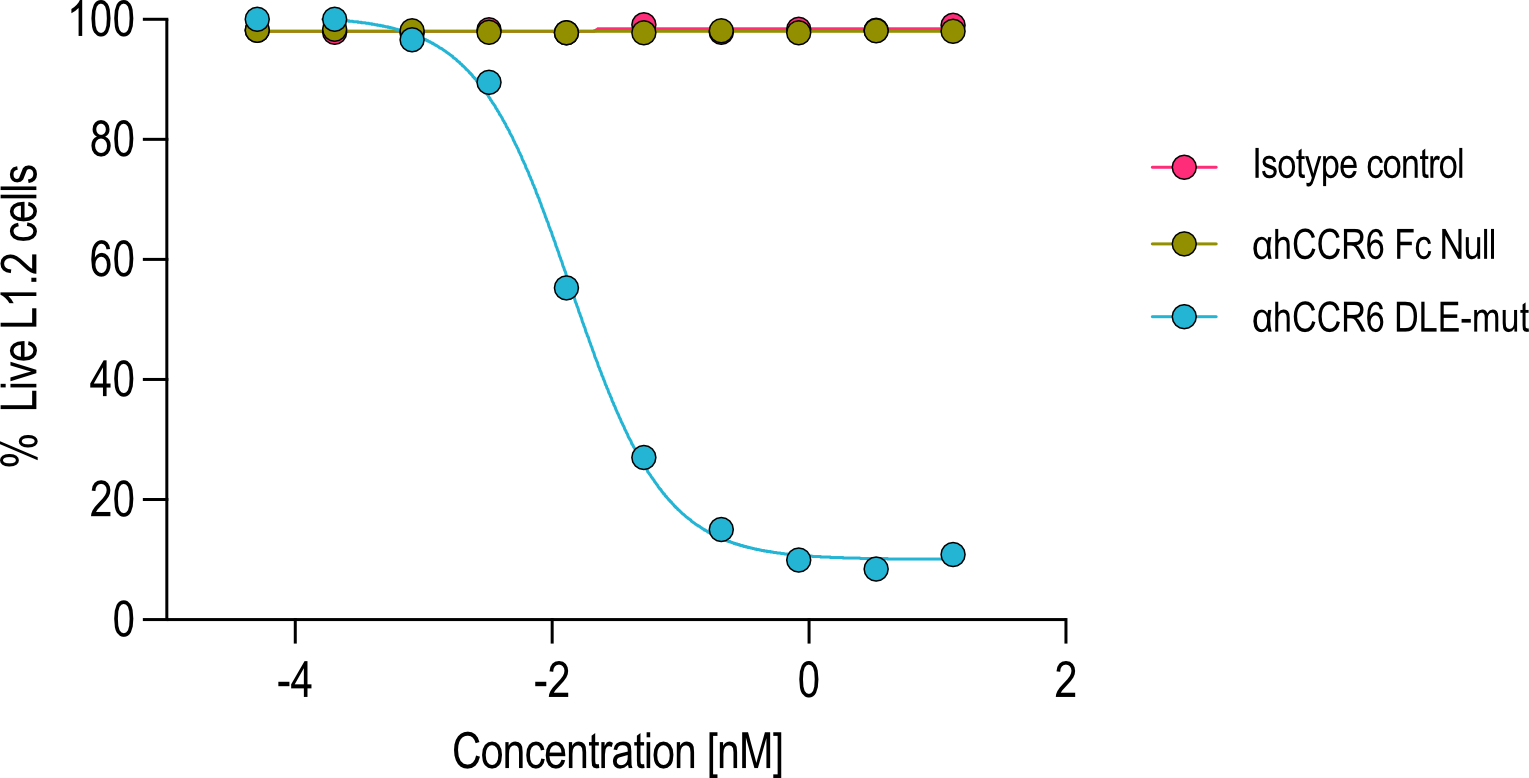

Supplement: Supplementary Figure 1 — Functional characterization of αhCCR6 mAb in NK cell-mediated cytotoxicity assay. Activated human natural killer (NK) cells (effector) were co-cultured with hCCR6-expressing L1.2 target cells at a fixed effector-to-target (E:T) ratio of 4:1. Cytotoxic activity was assessed in vitro using increasing concentrations of αhCCR6 DLE-mut, αhCCR6 Fc-Null, or human IgG1 isotype control mAbs. A dose-dependent enhancement of NK cell-mediated killing was observed with αhCCR6 DLE-mut mAb, demonstrating its functional potency and target specificity. [file Image1.tiff]

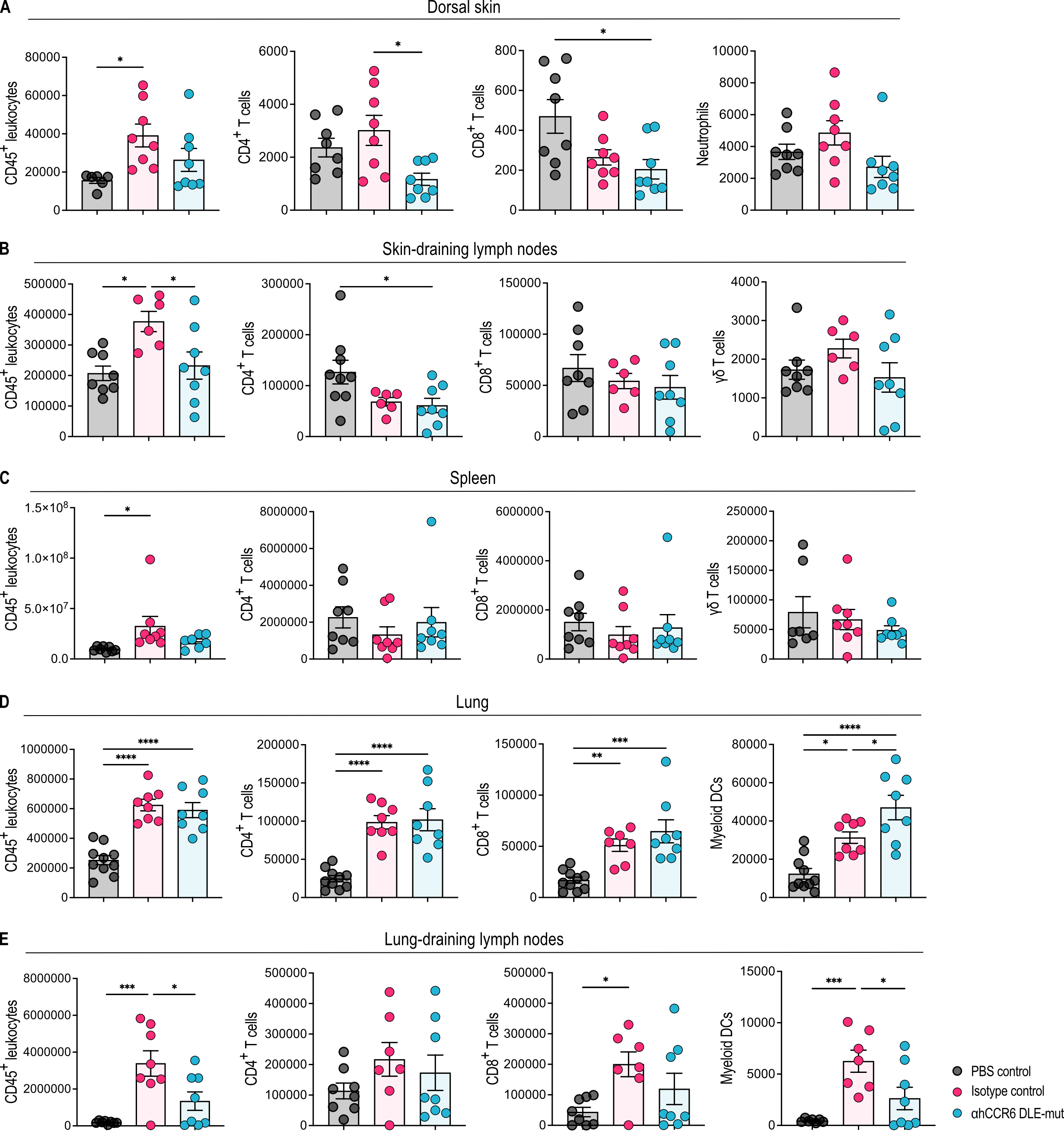

Supplement: Supplementary Figure 2 — Effect of αhCCR6 mAb on immune cell populations in the skin, lung, draining lymph nodes, and spleen of bleomycin (BLM)-treated mice. hCCR6-Tg/mCCR6-/- mice were subcutaneously injected with 100µg of Bleomycin or PBS (as control) for 28 days. On day 8, when dorsal skin thickening (~15%) indicated disease progression, and the onset of fibrotic signs (32, 33), mice were administered intraperitoneally (i.p.) a loading dose (25 mg/kg of body weight) of either αhCCR6 or an isotype control mAbs, followed by maintenance doses (5 mg/kg, i.p.) twice a week. At the end of the experiment, single-cell preparations were prepared from the dorsal skin, spleen and skin-draining LNs and analyzed by flow cytometry. The effect of αhCCR6 mAb treatment on the number of leukocyte subsets infiltration was assessed in: (A) Dorsal skin, (B) skin-draining LNs, (C) Spleen, (D) Lung, and (E) Lung-draining LNs. All data represented as means ± SEM; n = 6 to 8 mice for each group. Statistics were calculated using one-way analysis of variance followed by Tukey’s multiple comparison test. *P < 0.05, **P < 0.01, ***P < 0.001, ****P < 0.0001. [file Image2.tiff]

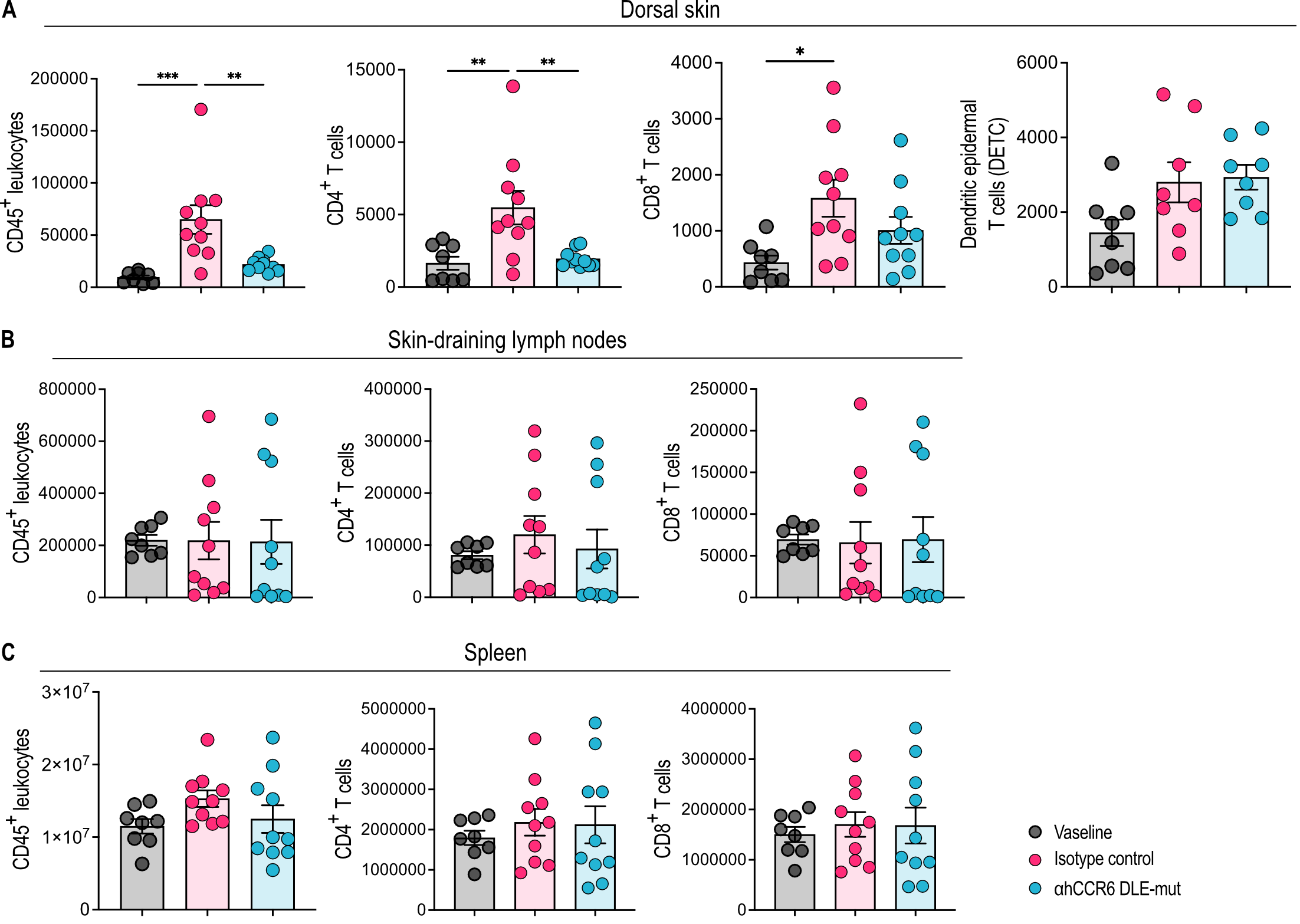

Supplement: Supplementary Figure 3 — Effect of αhCCR6 mAb on immune cell subsets in the skin, skin-draining lymph nodes, and spleen of imiquimod (IMQ)-treated mice. hCCR6-Tg/mCCR6-/- mice were topically applied with 20 mg imiquimod (IMQ) 5% cream or Vaseline (as control) for 7 consecutive days. On day 3, when dorsal skin thickening (~30%) indicated disease onset, mice were administered intraperitoneally (i.p.) a loading dose (25 mg/kg of body weight) of either αhCCR6 or an isotype control mAbs, followed by maintenance doses (5 mg/kg, i.p.) every other day for a week. At the end of the experiment, single-cell preparations were obtained from the dorsal skin, skin-draining LNs, and spleen and analyzed by flow cytometry. The effect of αhCCR6 mAb treatment on the number of leukocyte subsets infiltration were assessed in: (A) Dorsal skin, (B) skin-draining LNs, and (C) Spleen. All data represented as means ± SEM; n = 8 to 10 mice for each group. Statistics were calculated using one-way analysis of variance followed by Tukey’s multiple comparison test. *P < 0.05, **P < 0.01, ***P < 0.001, ****P < 0.0001. [file Image3.tiff]

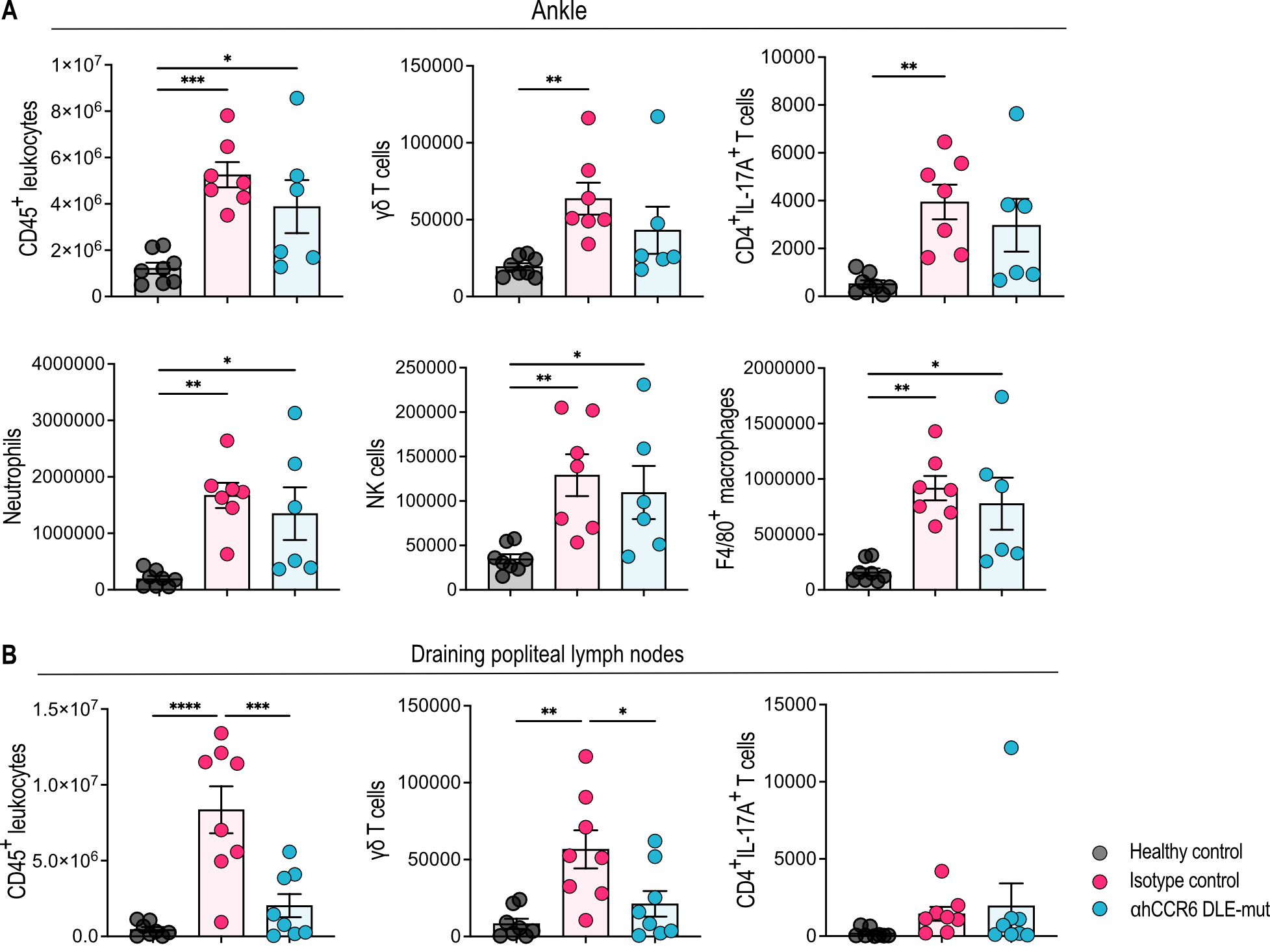

Supplement: Supplementary Figure 4 — Effect of αhCCR6 mAb on immune cell populations in the ankle and draining nodes in collagen-induced arthritis (CIA). hCCR6-Tg/mCCR6-/- mice were subcutaneously injected with an emulsion of 100 µg type II collagen (anti-CII) and 200 µg M. tuberculosis in Complete Freund’s Adjuvant (CFA) on Day 0 and given a booster injection of CII emulsified with Incomplete Freund’s adjuvant (IFA) on Day 21 to induce CIA. When mice exhibited swelling in ankle joints and front paws with an average cumulative clinical score of 4 (day 41), mice were treated with intraperitoneal (i.p.) injections of 5 mg/kg of body weight with αhCCR6 or isotype control mAbs, every other day for 3 weeks. At the end of the experiment, single-cell preparations from the ankle and draining popliteal LNs were prepared and analyzed by flow cytometry. The effects of αhCCR6 mAb treatment on the number of leukocyte infiltrations were assessed in: (A) Ankle and (B) Draining popliteal LNs. All data represented as means ± SEM; n = 6 to 8 mice for each group. Statistics were calculated using one-way analysis of variance followed by Tukey’s multiple comparison test. *P < 0.05, **P < 0.01, ***P < 0.001, ****P < 0.0001. [file Image4.tiff]
